# Comparative View of the State of Medicine in France, England, and Germany, from a Tour Made in Those Countries during the Year 1835

**Published:** 1837-04

**Authors:** 


### Art. X.

*Durstellungen und Ansichten zur Vergleichung der Medicin in Frankreich, England, und Deutschland, nach einer Reise in diesen Ländern im Jahre* 1835. Von Dr. Adolph Mühry, Practischem Arzte und Wundarzte in Hannover. *Mit zwei Plänen.—Hannover,* 1836. 12mo. pp. 283.

*Comparative View of the State of Medicine in France, England, and Germany, from a Tour made in those Countries during the Year* 1835. By Dr. Adolph Mühry, &c. *With two Plans.—Hanover,* 1836. 12mo. pp. 283.

In addition to medicine, Dr. Mühry has also touched upon the state of surgery and ophthalmology in the countries he visited. The work is concise, and written in a lively style, differing in this respect from the verbose phraseology and *infarcted* sentences with which German writers so frequently overwhelm the reader.

After taking a topographical survey of the hospitals and medical schools of Paris, London, and Edinburgh, the author ingeniously endeavours to prove that it is to the different meaning entertained of *inflammation* that we are to ascribe the fundamental differences between French and English medicine. In England, the doctrine of inflammation is more particularly associated with the principles of surgery; in France, with those of medicine: and this is true not only in point of theory, but (what is far more important) in actual practice. The development of the doctrine of inflammation as applied to surgery in England, is due to the labours of John Hunter; as applied to medicine in France, to M. Broussais.

"Hunter's admirable researches deserve our highest praise. While investigating profoundly the natural structure of animals and plants, he was not neglectful of medicine. Yet certainly, in expounding anatomy, both human, comparative, and pathological, and preeminently the processes of inflammation, he rather exalted the surgical branch of the subject than the medical. He clearly demonstrated, amid the confusion of conflicting opinions, that pus was not formed without preceding inflammation; applied with singular felicity and skill the processes of secretion, granulation, adhesion, and absorption, to surgery; and made the subject of inflammation the very centre point of all pathological enquiry. Hunter having thus rendered inflammation subservient to illustrate the consequences of wounds, and the condition of the blood and vascular system in its symptoms and terminations; and having pointed out the modifications it underwent in certain textures anatomically different, as likewise its specific nature, thereby contributed in a great measure to change the whole face of practical medicine in England."— . . . "His disciples, following the track thus plainly indicated, took for their groundwork the anatomy of regions, and viewing inflammation exactly as their master had unfolded it, and subsequently annexing to it the theory of irritation, promulgated by Sir A. Cooper, they thereby reduced the treatment of surgical diseases to such a pitch of simplicity and clearness as cannot fail to excite the wonder and emulation of other nations."

After the doctrine of inflammation had in this manner obtained a prominent place in science, its application in France took a somewhat different turn: there it ceased to maintain the surgical character it had acquired in England, and assumed the medical. This was mainly owing to the discoveries and doctrines of Bichat, Pinel, and especially of Broussais. He pushed to its maximum development that doctrine of inflammation to which the labours of Bichat had furnished the clue; and subsequently, as is well known, founded on its basis a new system of medicine.

"In looking at the state of medicine and surgery in England and France, it will be seen, in the former country, that surgery is in perfect correspondence with the sure and simple knowledge of *surgical* inflammation, while medicine is less associated with inflammation,—whether too little, is not easy to determine. In France, inflammation preponderates so much in medicine as to constitute an almost uniform local affection; while in surgery it is defective without doubt. Thus the medicine of England is not fettered by the unsettled notions of *medical* inflammation of the French; while the surgery of the latter stands in need of a more intimate acquaintance with, and adoption of, the definite *surgical* inflammation of the English authors."

A succinct account of Broussais' speculations closes with some allusions to the declining reputation of the Professor, and the little esteem now manifested towards them.

"In how much consideration Broussais has been held is well known. Now this is no longer the case. You see him seated in the chair of the vast amphitheatre of the Ecole de Médecine, arrayed in the black gown and red cap of the Faculty, and with blue spectacles on his nose; of a sudden his eye is fixed as the word *irritation* flashes across him; he becomes more and more excited, and at last turning to his auditors, who occupy little more than a single bench, exclaims with energy, '*Pourquoi donc, jeunesse, ne se trouve personne entre vous avec assez de bons sens pour s'opposer contre cette opinion de symptômes généraux, afin que la science marche, marche?*'"

The subjoined document from M. Charles Dupin, the distinguished statistician, may serve to corroborate the above statement as to the gradual declension of *Broussaisism.** Until the year 1823, France exported upwards of a million of leeches. Afterwards, she not only drained her own waters, but imported quantities from England and Germany, in order to meet the increasing demand; for, with Broussais and his followers, it was "*toujours les sangsues.*"

* Le Temps, 7 April, 1836.

| | Import. | Export. |
|---|---|---|
| 1820 | . . . . . . . . | 1,157,920 |
| 1823 | . . . 320,000 | 1,188,855 |
| 1827 | . . . 33,634,494 | 196,950 |
| 1833 | . . . 41,654,300 | 868,650 |
| 1834 | . . . 21,885,465 | 868,650 |

Dr. Mühry has formed a very just estimate of French therapeutics. It is remarkable that, with all the light to be derived from morbid anatomy, the treatment of disease should be so deplorably bad in that country.

"Therapeutics, the end and aim of all medical investigation, does not obtain that attention it justly demands in France. How very few internal remedies are permitted according to the dogmas of Broussais, is well known. It is long since the English physicians told their French *confrères* that they allowed their patients to die, while the latter imputed to the former that they killed theirs. When one hears the prescriptions ordered in the sick wards of a Parisian hospital, he is struck with astonishment at the meager diet and scanty medicine allotted to each individual. *Un quart d'une portion, un pain, un bouillon, décoction de riz, sirop de gomme,* constitute at once the nourishment of the patient and the *excipient* for his medicine." . . . "The supply of victuals is so inconsiderable, that many invalids, especially those suffering from chronic ailments, are heard piteously imploring the physician, while settling their diet-table for the day, to grant them a greater portion or small additional loaf of bread. Indeed, they may well complain of pain at stomach; hunger is the cause of it. The numberless potions forced upon them induces by and by squeamishness; food is loathed and rejected; and, when a cure is accomplished, it is rather through starvation, or withholding aliment, than by the employment of medicaments. *Lavemens* of various sorts are, on the other hand, in great repute; such as *lavements purgatifs, laxatifs, astringents, calmants, camphrés, de belladonne, oxymellés,* &c." . . "Every French practitioner is at any rate so far imbued with *Broussaisism* as to imagine the alimentary canal always more or less affected in disease, and hence is bound to abstain from the more efficacious internal remedies. Should neutral salts, calomel, opium, or antimonials be exhibited, it is merely for the sake of temporary trial; they soon give place to other things." . . . "The practitioner strives rather to procure a victory over the disease than the recovery of the patient; disregarding the general habit of body for a *localization.* With avidity will he abstract some *palettes* of blood from a subject extenuated by fasting and disease; stifling instead of cherishing the few remaining embers of life. It is thus manifest that, if French medicine be preeminent in diagnosis, based on anatomico-pathological research, it is very far behind in therapeutics."

Dr. Mühry has been at some pains in collecting materials for his chapter on English Medicine. He fairly gives us credit for being excellent in observing, in describing, and in treating disease. The several points touched upon are familiar to our readers; we shall therefore solicit attention to some matters of more novel interest.

The nasal catheter of Gensoul at Lyons seems to be an instrument of utility.

"It is remarkable how easily catheterism of the nasal canal can be effected by its means. The catheter is hook-shaped; the extremity, bent at a right angle, is about an inch in length, suited to the distance of the lower orifice of the nasal canal from the nostril, and likewise to the length and form of the canal, with a slight spiral turn. Conducted into the nostril, while the handle lies vertically upon the upper lip, it remains in the inferior meatus on the floor and outer walls; the point of the catheter will now be found anterior to the aperture of the canal, and a slight inclination outwards, together with a simultaneous and gradual elevation of the handle, causes the point to slip in; with a little assistance, the instrument passes up to the lachrymal sac. In the dead body, the point of the instrument may be seen protruding beneath the skin. On withdrawing the catheter, the handle is to be brought back to the vertical position with the upper lip. When we wish to procure mechanical dilatation, then is the method of arriving at the canal from below in every case preferable; no incision is required to be made; you are thus independent of the uncertain operation of puncturing from above inwards; a procedure which many eminent oculists hold in secret abhorrence. An additional advantage is, that injections, which, in certain morbid conditions of the mucous membrane of the canal, prove beneficial, can be thus efficaciously applied, and catgut bougies may be inserted in the same way. The introduction of Gensoul's nasal catheter is very simple. I have repeated the operation a number of times in succession on the dead body, and never once failed; and also practised it on the living subject in several instances; it did not occasion any considerable titillation."

Velpeau has recently introduced into practice a novel method of treating acute inflammation of the conjunctiva and sclerotic. It consists in covering the whole eye with a blister. He recommends you, before applying it, to exercise gentle friction on the skin of the eyelids with a fold of linen moistened in vinegar. The epispastic plaster must be large enough to embrace the whole surface of the orbital cavity: it is not necessary to shave the eyelids or eyebrows beforehand. The eyelid being closed, the blister is put on, and maintained *in sitû* by charpie and a bandage; the charpie is useful for filling up interstices. On the following day it is removed, and the vesicated surface bathed with tepid water. In two or three days the surface heals, and the inflammatory symptoms subside.

We shall conclude our notice of this little work with a few remarks taken from it, on the speculative views of some of the author's countrymen.

Towards the close of the last century, that mysticism which pervaded German philosophy extended itself to medicine. A sort of triple alliance ensued betwixt medicine, philosophy, and poetry. All was ideal, nothing positive. Apollo resumed his double reign: physiology was expounded in stanzas, pathology became the theme of heroic metres. The methods of research were synthetical rather than analytical; general rather than special. In comparing the relation of objects, the most far-fetched analogies were sought after. Mysterious symbols were introduced, in order to characterize unknown relations. That the Germans were at this time not so much philosophers as philosophical poets, is evident from the circumstance of their pursuing intellectual beauty rather than scientific truth. They were in quest of the shadow, not of the substance. They dwelt so much in the regions of fancy, that the epithet "*geistreich*" (spiritual,) denoted the highest praise. An idea became intrinsically valuable, independently of its truth or applicability; and colossal theories were constructed upon the most unstable bases. Among other innovations, a new nomenclature started up, with terms borrowed from "their philosophy," whose meaning was intelligible only to the initiated few. During this period, which well deserves the appellation of *the ideal*, medicine in Germany was comparatively stagnant; for the time which might have been devoted to extend the boundaries of practical knowledge, was spent in fruitless speculation. Meantime many important discoveries were made in France and England. There the votaries of science, following the dictates of reason, not the vagaries of imagination, contributed eminently to improve various branches of the healing art.

One of the most remarkable of these *ideal* systems now prevalent is that of Jahn and Carl Hoffmann, of which we purpose laying an account before our readers, in a review of the curious work of the former author, entitled "System der Physiatrik." The fundamental principle of this system is, that all the classes of diseases in man are analogous to, if not identical with, the natural conditions which distinguish particular classes of animals. Thus, rheumatism is analogous to the nature of insects; inflammation to that of birds; dropsy to that of hydatids, &c.

Another of the Ideal or Poetical systems, is that of Schönlein. This physician was formerly professor of pathology at Würzburg, and is now of Zurich. His system is called the *natural system*, and is in great vogue. Less purely speculative than many other German systems, it pretends to consider medicine as a natural science. There are, however, certain essential points of difference. Natural science looks upon disease as an aberration of the normal mode of vitality; in other words, as a morbid condition. The scheme in question, on the other hand, perceives in disease not a deviation from the healthy standard, but a peculiar vitality. Disease is in itself an organism. The school of Schönlein goes directly to the malady; it describes without explaining it. It attempts no definitions, but, as in the instance of organisms, only descriptions; and, assuming a poetical licence, it transforms the disease into a plant. In order, therefore, to sustain and follow out the comparison, there has emanated not merely a natural, but a *botanical* system. Hence diseases are distinguished into families and species; we find phanerogamic and cryptogamic ailments; mention is made of the relations of morbid vitality, and of the physiology of disease,—of the natural history of disease,—of the seeds of disease,—of its geographical distribution,—of the misshapen forms, &c.; precisely as in botany. The contrasting of plants and diseases affords ample scope to those of an ingenious turn. Thus, the accidental tissues or parasites may be said to bear a certain similarity to plants; after them, the exanthemata, and process of inflammation. But, when such comparisons, which ought to be taken only in a figurative sense, are made subservient to the study and arrangement of nosology, they will prove eminently calculated to mislead. Facts are neglected, and habits of inaccurate reasoning acquired. False premises are assumed, and erroneous conclusions follow. In common speech, flowers are often employed metaphorically to denote the abstract qualities of objects; in the above system, however, they form a constituent part. Thus measles is called the inflorescence of catarrh, and scarlatina of erysipelas. Among cutaneous affections, we find the *impetigines* botanically placed according to their anatomical form. Those whereof the type is indistinctly marked, are designated *crypto-impetigines;* in others the elevations of the cutis are *impetiginous fruits*, distinguished into the pericarp and proper fruit. The form of herpes is thus described: "a common pericarp, fruit collected in groups, mostly vesicular." The tribe of *psoræ* has for its distinctive character a separate *fruit-ground*, with solitary fruit, &c.

In his chapter of concluding comparisons, Dr. Mühry observes, that his countrymen are unquestionably far advanced in the organization of schools and universities; in state and legal medicine; in the obstetric art, and in ophthalmology. He says, "The actual state of science in other lands is, in England and France, not so well known as in Germany. In this respect the French betray a harmless ignorance, the English a morbid indifference. Among either it is only the junior branches of the profession who cultivate foreign medicine. The French are more familiar with English medicine than with German, and conversely in England. The Germans are equally well acquainted with the medicine of each country. In England, the hospitals for the sick were, in the first instance, subservient to the instruction of the medical student; lectures on the institutes of medicine were subsequently superadded. In Germany, on the contrary, theoretical lectures were at first delivered in the universities, and hospitals afterwards annexed for purposes of clinical illustration." Dr. Mühry remarks, that the relative estimation of the medical faculty, like apothecaries' weight, is nearly the same in every civilized country. But our general practitioners are for the most part of a lower grade than their professional brethren in France and Germany. He contends that a French practitioner directs his attention more to the disease than the patient; an English practitioner, disregarding the case before him, reflects on some other in his past experience; while the German practitioner entertains the wholesome doctrine thus expressed by Hufeland, "generalize the disease, but individualize the patient." The French generalize the patient, the English individualize the disease.

---

